# Neuropathic Pain Relief after Surgical Neurolysis in Patients with Traumatic Brachial Plexus Injuries: A Preliminary Report

**DOI:** 10.1155/2022/5660462

**Published:** 2022-08-02

**Authors:** Armando Armas-Salazar, Noe Téllez-León, Ana Isabel García-Jerónimo, Francisco Alberto Villegas-López, José Luis Navarro-Olvera, José Damián Carrillo-Ruiz

**Affiliations:** ^1^Mexican Faculty of Medicine of La Salle University, Mexico City, Mexico; ^2^Postgraduate Department, School of Higher Education in Medicine, National Polytechnic Institute, Mexico City, Mexico; ^3^Functional & Stereotactic Neurosurgery & Radiosurgery Service, General Hospital of México, Mexico City, Mexico; ^4^Physical Medicine & Rehabilitation Service, General Hospital of México, Mexico City, Mexico; ^5^Faculty of Health Sciences Anahuac University, Mexico City, Mexico; ^6^Research Direction, General Hospital of Mexico, Mexico City, Mexico

## Abstract

**Objective:**

To evaluate the usefulness of surgical neurolysis for neuropathic pain relief in patients with posttraumatic brachial plexus injury (BPI).

**Methods:**

A prospective, longitudinal, nonrandomized, self-controlled before and after study was performed to evaluate the pain changes according to their intensity using the Visual Analogue Scale (VAS), and the sensory recovery after surgery using the British Medical Research Council (BMRC) scale for sensory recovery. To establish significant changes, a paired *T*-test was performed, and in order to determine the magnitude of these changes, an effect size was measured. *α* = 0.05.

**Results:**

Ten patients were included with an average follow-up of 61.9 ± 53.62 months. The main mechanism of injury was vehicular trauma (70%). A significant decrease in pain after the surgical intervention was observed resulting from an average preoperative state according to VAS of 8.4 ± 1.58, to a postoperative state of 3.4 ± 3.27 (59.52%, *p* = 0.005, Δ = 1.572), added to a mean sensory improvement (25%) from 2.8 ± 1.62 to 3.5 ± 0.97 after surgery according to BMRC, without statistically significant changes (*p*=0.062), showing a moderate effect size (Δ = 0.413). Almost all patients showed improvement in the continuous and paroxysmal pattern of pain. No postoperative complications were observed. *Discussion*. These results suggest that in cases of BPI that originates from a compressive syndrome secondary to the posttraumatic fibrosis that surrounds the nerve structures causing strangulation and inducing hypernociception, the use of surgical neurolysis is an appropriate alternative for patients with medically refractory neuropathic pain.

## 1. Introduction

Neuropathic pain caused by a lesion or disease of the somatosensory nervous system can result from nerve compression in posttraumatic brachial plexus injuries (BPI) produced by the connective tissue (fibrosis) that surrounds the nerve structures, generating a compressive phenomenon that causes strangulation of the nerve, inducing hypernociception mediated by inflammatory mediators [1]. Therefore, surgical management is helpful when the underlying cause is identified. The literature on the study of BPI reflects that the attention of the clinical outcomes reported in the various articles on this pathology is focused on evaluating the motor component [2], displacing the study of other associated comorbidities such as pain, despite being a highly prevalent condition [3]. The few reported studies on surgical pain management in BPI predominantly evaluate the effectiveness of dorsal root entry zone lesions, DREZotomy [4]. Conversely, regarding the use of neurolysis in pain management, there are no articles that aim to evaluate the specific role of pain recovery in BPI after surgical neurolysis alone. Anecdotical cases of pain recovery after surgical neurolysis are reported [5, 6]. In relatively recent years, an article published by Bonilla G. et al. (2011) evaluated pain recovery in different surgical techniques (including neurolysis), showing pain relief in some patients without precise outcome measures [7]. The most recently published article on this topic was published by Morgan R. et al. (2020), in which they described the improvement of pain in 21 patients with BPI, secondary to management with external neurolysis and open fasciotomy. However, this study focused on the study of distal lesions and not proximal to the brachial plexus [8]. Therefore, there is a lack of information that describes the role of surgical neurolysis in pain management in BPI.

Surgical neurolysis is a technique that began to be used in World War by exploration of the wound and debridement of the affected nerve. There are two types of surgical neurolysis, both with the purpose of decompressing the affected nerve structure; the internal neurolysis consists of making multiple longitudinal cuts along the epineural area of the affected nerve structure, and the external neurolysis, which consists of dissecting the connective tissue that surrounds the injured nerve structures, lysing the adhesions formed in the compartment. The dissection is usually careful and slow to protect the underlying structures and preserve the continuity of the nerve [9]. In 1996, Clarke. et al. [10] reported a study where they determined that neurolysis did not represent significant clinical changes compared to spontaneous recovery in patients with BPI, being a transcendent study because it ended up defining neurolysis is an ineffective technique for the management of BPI. This argument added to the popularization of the study of other techniques such as nerve graft, nerve transfer, and muscle/tendon transfer, led to the abandonment of surgical neurolysis, decreasing the number of clinical studies carried out on this technique [2], highlighting the need to re-evaluate the role of surgical neurolysis in patients with traumatic BPI, and assessing whether it is feasible to reposition it as a useful technique. For these reasons, the aim of this study was to clarify the involvement of neurolysis and its repercussion in neuropathic pain amelioration.

## 2. Materials and Methods

### 2.1. Study Design and Participants

A prospective, longitudinal, nonrandomized, self-controlled before and after study was performed according to Consolidated Standards of Reporting Trials (CONSORT) 2010 statement for reporting nonrandomized studies [11], to evaluate pain and sensory recovery in a group of patients with neuropathic pain as the main clinical manifestation secondary to BPI of traumatic origin after surgical management with neurolysis. The first measurement was done before the surgical intervention, and the second at the last time of follow-up after surgical management. The study was carried out in the peripheral nerve clinic of the neurosurgery department of the General Hospital of México. The study protocol was approved by the Ethics and Research Committees and was conducted in accordance with the Declaration of Helsinki. Written informed consent for surgery was obtained from each subject. According to the eligibility criteria, all adult patients (18–70 years) of both genders, with BPI of traumatic etiology, high level of injury (proximal third of the upper extremity), and refractory neuropathic pain (medical treatment with at least 2 different analgesic drugs during three months of management) were included. Those patients with avulsion, preganglionic injury, precervical lesion, nerve transection, and absence of compressive neuropathy (determined through a preoperative electromyography study determined by a neurogenic pattern with positive fibrillations, polyphasic units, and an increase of firing rate) were excluded. The selection process of the patients is shown in Supplemental Figure 1.

### 2.2. Data Collection

The data extraction was focused on collecting information on the demographic aspects (age, gender), etiology, anatomical location of the injury, affected side, interval injury-surgery, and average follow-up (the period of time established as follow-up corresponds to the last moment in which neuropathic pain was clinically evaluated). The clinical evaluation of the patients was focused on collecting data corresponding to the preoperative and postoperative state of two clinical components (pain and sensory). It was decided to assess pain according to its intensity using the Visual Analogue Scale (VAS), the most validated measurement method [12], and the sensory component was assessed using the British Medical Research Council (BMRC) scale for sensory recovery, a scale that evaluates deep and superficial cutaneous sensibility (Grade 0 represents that there was no improvement, and Grade 4 a complete recovery) [13]. The main focus of the study was the study of pain recovery after surgical neurolysis. However, it was decided to add the evaluation of the sensory component to the analysis because it is intended to establish whether there is a relationship between the sensory component and pain recovery. The pain was evaluated at 2 moments, before surgery and on the last time of patient follow-up (61.9 ± 53.62 months), obtaining a long-term evaluation.

### 2.3. Surgical Technique

Approaches located in the supraclavicular fossa were performed, through a “V-shaped” incision following the posterior border of the sternocleidomastoid and the inferior border of the clavicle, the *platysma* aponeurosis was lifted, taking care of the underlying external jugular vein (ligating it on occasions to avoid a nearby injury). Subsequently, the omohyoid muscle (guiding point of the approach) was delimited, moving it with a surgical rubber band without the need to section it. Under the thickness of the adipose tissue, the transverse cervical artery was identified, which was ligated and sectioned. Subsequently, a dissection of the anterior interscalene triangle aponeurosis was performed (preserving the integrity of the phrenic nerve that runs under the anterior scalene aponeurosis). In some cases, the ascending cervical artery could be seen parallel to the phrenic nerve, whose integrity was always preserved. Thereafter, the exposure of the upper trunk (C5–C6) was performed, which was surrounded with a surgical band for manipulation and cranial displacement for the exposure of the middle (C7) and lower trunks (C8-T1). All the trunks of the brachial plexus were explored deeply, no muscles were sectioned, and all surgical corridors (between nerves, muscles, and aponeurosis) were used to perform the procedure. External surgical neurolysis was realized; it consisted in releasing the tight fascia, muscle, and tendon that are compressing the nerve and cutting out the scar tissue around the nervous structures, to avoid the compressive phenomena caused by the scar tissue that was adjacent to the injury. The extent of neurolysis was determined by the compression sites observed at the time of surgery. All the procedures were carried out by the corresponding author.

### 2.4. Statistical Analysis

Descriptive statistics were used to analyze the patients' characteristics by calculating the mean and standard deviation of the demographic (age) and procedural factors (interval injury-surgery and follow-up), as well as frequencies to describe the other characteristics (gender, mechanism of injury, location of injury, and side affected).The clinical changes were also represented as median and maximum and minimum ranges, due to the nonparametric distribution of the sample. However, it was decided to perform the representation with means and standard deviations because it is usually the most frequent form as reported in the literature. To establish significant changes after surgery in pain and sensory outcomes, a paired *T*-test and a Wilcoxon signed-rank test were performed to establish the relationship between the preoperative and postoperative changes. In order to determine the magnitude of these changes, an effect size of postoperative outcomes in pain and sensory components was measured, the effect size was calculated using the Cohen's *d*, and recalculated considering the correction coefficient for small sample sizes to avoid overestimating the effect measure. A sample size calculation was carried out (Supplemental Figure 2). Statistical comparisons of the outcomes involved were performed using SPSS 25.0 for Windows software (SPSS, Inc., Chicago, IL), where a *p* value < 0.05 was considered significant.

## 3. Results

**Figure 1:**
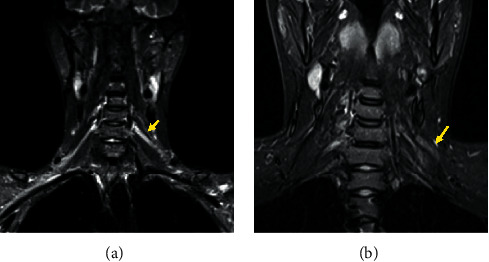
T2 weighted MRI of the brachial plexus showing the onset of the compressive phenomenon after post-traumatic injury, where the left panel shows an initial inflammatory process at an early stage of injury and the right panel image shows the development of fibrosis at a later stage. (a) The arrow points the swelling of the root with increased signals. (b) The image shows a fibrotic process located in the left C5 to C7 brachial plexus trunks (arrow), secondary to the traumatic injury, manifested as pain, symptom explained by the connective tissue that surrounds the nervous structures, generating a compressive phenomenon that originates strangulation of the nerve, inducing hypernociception.

A total of 10 patients met the inclusion criteria for the analysis. According to gender, the percentage of males predominated (80% of males). The average age of the population at the time of the injury was 34 ± 16.85 years old. The main mechanism of injury was vehicular trauma (Topside motorcycle crash; motorcycle suddenly decelerates and rider flips over the handlebars) in 7 cases, followed by industrial trauma, stab injury, and hit by a vehicle (10% each). Roots injuries from C5 to T1 were present in only 3 patients. Radiologic features in MRI represented the compromise evolution of traumatic BPI in the included patients and are shown in Figure 1. Regarding the procedural characteristics, the time interval that existed between the injury and the performance of the surgery was 10.6 ± 5.46 months on average. The patients had an average follow-up of 61.9 ± 53.62 months.  Table 1 shows the characteristics of the included patients.

### 3.1. Outcomes

**Figure 2:**
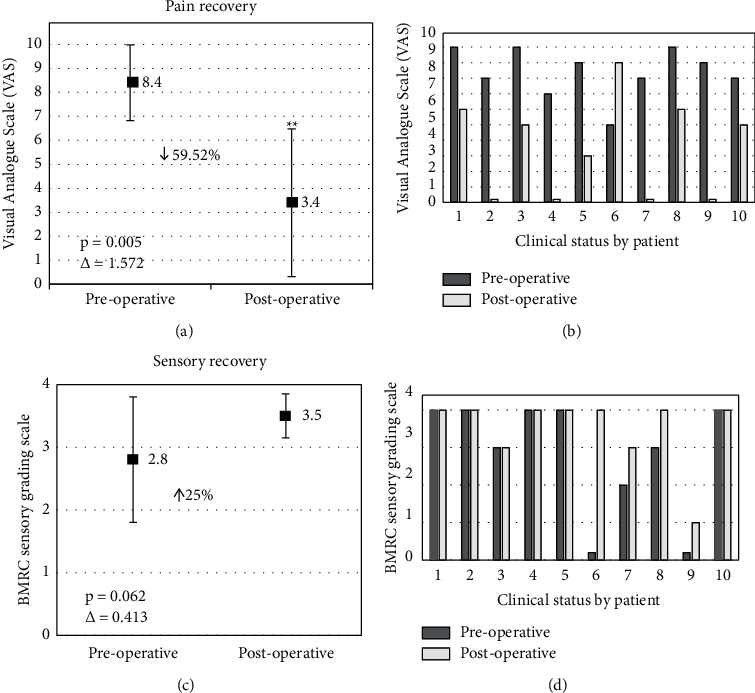
Clinical outcomes. (a) Pain global recovery showed a significant decrease (*p*=0.005) in postoperative pain intensity with a large effect size (∆ = 1.572). (b) The changes in pain intensity highlight that patient number 6 showed a worsening of his clinical situation after surgical intervention. (c) There was a recovery of sensory function in terms of discriminative touch and deep sensitivity of 25%. However, these changes were not significant (*p*=0.062). (d) The individualized analysis demonstrated preservation of the sensory status in 50% of the patients, whereas the rest showed an improvement.

The results of the surgical intervention in the motor and sensory components are shown in Figure 2. Clinical assessment one week after the surgical intervention showed a decrease of more than 50% according to the VAS, with respect to the preoperative status. The short-term evaluation was not considered for the statistical analysis because patients could tend to over-/under-estimate the improvement, leading to analyzing imprecise measures. In long-term follow-up (61.9 ± 53.62 months), a relevant decrease in pain after the surgical intervention was observed, resulting from an average preoperative state according to VAS of 8.4 ± 1.58, to a postoperative state according to VAS of 3.4 ± 3.27, these changes being statistically significant (*p*=0.005), which showed a large effect determined by Cohen's *D* (Δ = 1.572). With the aim of evaluating if the pain outcomes were secondary to an impact on the sensory component, it was decided to evaluate the sensory state before and after surgery, showing that the mean preoperative state according to the BMRC was 2.8 ± 1.62, changing to a postoperative state of 3.5 ± 0.97, resulting in a trend towards improvement in postoperative sensory component, without these changes being statistically significant (*p*=0.062), showing a moderate effect determined by Cohen's *D* (Δ = 0.413). Therefore, it was assumed that the surgery did not have a negative impact on the sensory component. All patients included before surgery were under pharmacological management (several neuromodulators and antidepressants) for their original neuropathic pain control, only 3 patients after surgery received pharmacological management: patient number 2 received tramadol and acetaminophen (400 and 2,250 mg/day, respectively), patient number 3 received gabapentin (250 mg/day), and patient number 7 received tramadol and acetaminophen (100 and 750 mg/day, respectively). Seventy percent of the patients did not require after surgery pharmacological treatment. Regarding the surgical intervention, almost 9 patients showed improvement in the continuous and paroxysmal pattern of neuropathic pain after surgery without predominance of one component over another. No postoperative complications were observed. Surgery did not affect motor function, even the patients presented an improvement in strength after the intervention (the discussion of these results is the objective of another study).

## 4. Discussion

This study showed that 9 patients (90%) had a decrease in pain (patients presented with preoperative pain equal to or greater than 7), where four patients (40%) presented a complete pain intensity reduction at the last follow-up (VAS = 0), with an overall decrease of 59.52%, statistically significant changes (*p* = 0.005), and an important clinically significant improvement (∆ = 1.572) after surgical neurolysis. Without showing an impact on the sensory component, there was an improvement of 25% without statistically significant changes (*p* = 0.062) but with a moderate effect size (∆ = 0.413). On the other hand, an aspect to highlight about patient number 6 (the only patient who worsened after the intervention Figure 2 c)) is that he was the patient with the lowest pain status of the group at the preoperative moment (VAS = 5); therefore, we assume that the intervention is more recommended in those cases where the pain is more severe. It is imperative to evaluate the repercussion that the symptom has on the patient's quality of life since apparently, this technique could be more effective in patients with severe pain. Surprisingly, despite the pain being much worse at long-term follow-up compared to the preoperative state, this patient had the most dramatic sensory recovery of all the patients in the series (no sensory function prior to surgery to full recovery of sensation at long-term follow-up), a phenomenon possibly explained by a masked process conditioned due to the absence of sensory function. In addition, patient number 6 is the patient with the longest interval injury-surgery (20 months); however, patient number 2 also had a prolonged injury-surgery interval (19 months) showing complete recovery, so this characteristic is controversial regarding the effectiveness of the technique. According to the results observed in patient number 6, we can establish that surgery is probably more effective in severe neuropathic pain (≥7 according to VAS), and the injury-surgery interval influences in some way the outcomes, as has been reported in previous studies [14].

In accordance with the findings, we can suggest that the clearest indication for the surgical management of neuropathic pain in patients with BPI is the presence of severe neuropathic pain (≥7 according to VAS) refractory to medical treatment, associated with a compressive syndrome secondary to a traumatic process; observations explained because the trauma conditions a local inflammatory process that leads to the development of fibrosis through the activation of fibroblasts, resulting in increased collagen synthesis and accumulation of thin and disorganized collagen fibers [15]. This fibrotic process produces a scar tissue that surrounds the affected nerve structures, generating a compressive phenomenon (detectable by preoperative electromyography) that results in strangulation of the nerves depriving it of blood flow, and induces neuropathic pain through a marked and long-lasting mechanical hypernociception acting by inflammatory mediators mainly related to neurotrophic factors such as nerve growth factor, neurotrophin-3, and glial cell line-derived neurotrophic factor [16]. Moreover, brain-derived neurotrophic factor is produced from sensory neurons and plays a critical role in mediating the transition from acute to chronic pain [17]. Therefore, surgical neurolysis allows these nervous structures to be released by separating the surrounding tissues, solving the upregulation of these inflammatory mediators that causes chronic neuropathic pain.

Early evaluations to establish a temporary relationship between surgery and symptom improvement have the limitation of not being able to define the effect; this is because patients over-/under-estimate their symptom relief while they are healing during the first few days/weeks after surgery [18]. For this reason, the evaluation at the first postoperative week was not considered in the analysis. In order to avoid this bias caused by the early appreciation of the effect, it was decided to consider the last time of follow-up for each of the included patients, obtaining a mean follow-up time of 61.9 months (from 12 months to 191 months). This method allows observing the effect adequately, as well as the effectiveness was related to the surgical procedure in the long term, and the benefit was significant regardless of the temporal context of the evaluation. The literature reflects that surgical intervention in patients with BPI totally changes the natural history of the disease; so regardless of the temporal context in which the measurements were made, it is clear that the changes are attributed to the intervention [19].

The neuropathic pain of the included patients consisted of the presence of pricking, tingling, electric shocks, burning sensations, and pain evoked by touching. The study focused on analyzing the decrease in pain intensity since it is the one most associated with an impact on quality of life. Pain after BPI is generally characterized by 2 main different components: paroxysmal (electrical shooting-like) pain, and continuous (burning) pain [20,21]. The patients included in the study showed improvement of both components, the continuous and paroxysmal pattern after surgical intervention. These outcomes were evaluated qualitatively because there are no standardized clinimetric scales for their analysis. The paroxysmal pattern is originated from the deafferented posterior spinal horn neurons, and the continuous pattern comes from the thalamus [22]. On the other hand, patients with neuropathic pain also had sensory deficits as a consequence of their nerve injury. These sensory abnormalities could be presented alone or accompanied by neuropathic pain. The BMRC scale for sensory recovery modified by Mackinnon and Dellon was used to assess the sensation function where discriminative touch was evaluated, and the deep and superficial cutaneous sensation [13].

One of the great advantages of the study is that the patients studied were a highly homogeneous group (trauma patients) with strict inclusion criteria. In addition, an adequate calculation of the sample size was made (Supplemental Figure 2). Although our study only included 10 patients, the effect size observed in a previous study on the use of surgical neurolysis in distal brachial plexus injuries showed a decrease in pain with an effect size of 1.606 (Morgan et al.(2020) [8]), gave our study a statistical power of 99%, so the external validity of the study is appropriate, considering the recommendations for pilot studies [23,24]. However, we consider that it is a preliminary study because it would be convenient to study more to test the feasibility of the procedure. Conversely, we consider that the main limitation is related to the study design, where randomization, blinding, and comparison with another standard have not been performed. However, it can be considered a self-controlled study because the surgical intervention totally changes the history of the disease [25], so that taking pre- and postoperative measurements allows to reliably observe the magnitude of the changes, without conferring negative ethical implications that will limit the development of the study, such as using a control group to which therapeutic management is not offered. It is also relevant to mention that the included patients presented lesions in different regions, where the most frequent location was in the C5–C7 roots (60%). Despite improvement being observed in all injury patterns, it would be appropriate to validate in subsequent studies whether the clinical response is influenced by the injury pattern. Relative to the severity of the injury, this technique was performed in patients with preservation of nerve continuity. Therefore, the included patients presented a homogeneity in terms of injury severity.

An exhaustive search has been made in the literature on the subject, in order to establish the previous antecedents published about the usefulness of surgical neurolysis for the relief of neuropathic pain in patients with BPI. An advanced search in PubMed was performed using the MeSH terms, “Brachial plexus neuropathies,” “Surgery,” and “ Pain,” obtaining a total of 108 results from 1951 to 2021. The selected articles were those clinical studies written in English that mentioned the outcome of pain after the surgical management with neurolysis in patients with BPI. A total of 4 articles published between 1977 and 2020 were selected for the analysis. Data extraction was focused on determining the preoperative and postoperative state of pain according to VAS, the existence of statistically significant changes, as well as the percentage of improvement of the patients in terms of pain by calculating the delta of the pre- and post-operative pain state. The results of the literature review are shown in Table 2, demonstrating a clear improvement in pain of 39.41% after surgical neurolysis, which according to Farrar J. et al. (2001) is considered a clinically significant response to treatment [12].

Surgical neurolysis shows an important relief of severe neuropathic pain in those patients with BPI who manifest a compressive syndrome of traumatic origin; these findings force us to think if we are adequately perceiving the effectiveness of surgical neurolysis in contemporary times, which years ago seemed to be a technique comparable to spontaneous recovery, a clear misconception. It is necessary to perform a complete clinical trial with a well-randomized, well-controlled, and well-powered design, in order to evaluate the benefits of this technique even in other clinical components such as motor recovery. Nevertheless, this study is a relevant precedent to reveal the possible indications for pain relief that surgical neurolysis has in the management of posttraumatic BPI, a surgical technique displaced and forgotten over the years.

## Figures and Tables

**Table 1:** Demographic, procedural, and clinical characteristics of the included patients.

Number of patients (gender)	Age	Mechanism of injury	Location of injury	Side affected	Interval injury-surgery (mos)	Follow-up (mos)	Neuropathic pain (VAS)		Sensory recovery (BMRC)	
							Preop	Postop	Preop	Postop
1 (M)	69	VT	C5-C6	L/R	12	37	10	6	4	4
2 (F)	21	VT	C5-C6-C7	R	19	60	8	0	4	4
3 (M)	33	HV	C7-C8-T1	R	6	191	10	5	3	3
4 (M)	20	VT	C5-T1	R	7	57	7	0	4	4
5 (M)	21	VT	C5-T1	L	9	40	9	3	4	4
6 (F)	27	VT	C5-C6-C7	L	20	72	5	9	0	4
7 (M)	43	VT	C5-C6	L	6	108	8	0	2	3
8 (M)	22	IT	C5-C6-C7	R	14	12	10	6	3	4
9 (M)	56	VT	C5-C6	R	8	24	9	0	0	1
10 (M)	28	SI	C5-T1	R	5	18	8	5	4	4
										
^*∗*^Mean (SD)	34 ± 16.85				10.6 ± 5.46	61.9 ± 53.62	8.4 ± 1.58	3.4 ± 3.27	2.8 ± 1.62	3.5 ± 0.97
Median (min-max)	27.5 (20 - 69)				8.5 (5 - 20)	38.5 (12 - 191)	8.5 (5 - 10)	4 (0 - 9)	3.5 (0 - 4)	4 (1 - 4)

VAS: visual analogue scale. BMRC: British Medical Research Council sensory grading scale. VT: vehicular trauma. HV: hit by a vehicle. IT: industrial trauma. SI: stab injury. SD: standard deviation. ^*∗*^The data were represented as mean and standard deviation despite being a nonparametric sample because in the literature they are usually represented in this manner.

**Table 2:** Surgical neurolysis for pain improvement in BPI: literature review.

Authors	Year of publication	Sample size (n)	Follow-up (months)	Surgical techniques	Pain VAS (preop)	Pain VAS (postop)	Statistical analysis (*p* value)	Percentage of improvement
Current Study	2021	10	61.9 ± 53.62	N	8.4 ± 1.58	3.4 ± 3.27	*p*=0.005	59.52
Morgan R. et al. [8]	2020	21	6 ± NM	N + OF	6.4 ± 2.50	2.0 ± 2.50	*p*=0.010	31.25
Bonilla G. et al. [7]	2011	51	6 ± NM	N, NG, NT	9.1 ± 0.20	2.5 ± 0.20	*p*=0.001	27.47
Narakas A. [6]	1978	NS	132 ± NM	N, NG, NT	NS	NS	NM	^*∗*^Improv.
Millesi H. [14]	1977	1	24 ± NM	N	NS	NS	NM	^*∗*^Improv.
								
Mean value ± SD			45.98 ± 53.22		7.97 ± 1.40	2.63 ± 0.71		†39.41

VAS: visual analogue scale, N: neurolysis, OF: open fasciotomy, NG: nerve graft, NT: nerve transfer. NS: not specified. NM: not measured. ∗Pain improvement referred by the author. †A reduction in pain greater than 30% can be considered as a clinically significant response to treatment [12].

## Data Availability

The data that support the findings of this study are available on request from the corresponding author, José D. Carrillo-Ruiz.

## Supplementary Materials

Supplementary Materials
*Supplementary 1. Supplemental Figure 1*. CONSORT trial flow diagram. ^*∗*^Injury located in the proximal third of the upper extremity (before reaching the proximal third of the humerus). †Pain refractory to medical treatment with at least 2 different analgesic drugs during three months of management. ^‡^Compressive origin was determined through a preoperative electromyography study determined by a neurogenic pattern with positive fibrillations, polyphasic units, and an increase of firing rate. *Supplementary 2. Supplemental Figure 2*. A sample size calculation was carried out using the program: *G*^*∗*^ Power 3.1.9.7 for Windows XP. The sample size calculation was performed according to effect sizes for a Wilcoxon signed-rank test, using the results reported by Morgan R. et al. (2020) [8], study where they evaluated the effects of surgical neurolysis and open fasciotomy for pain relief in 21 patients with distal BPI, reporting changes in pain intensity according to VAS of 6.4 ± 2.5 2 ± 2.5 postoperatively, resulting in an effect size of 1,606. The sample size analysis resulted in a total number of required patients of 10 for a statistical power of 99%. *s*
