# Supplementary figures and images for: Neuropathic Pain Relief after Surgical Neurolysis in Patients with Traumatic Brachial Plexus Injuries: A Preliminary Report
Source: Pain Res Manag. 2022 Aug 2;2022:5660462. doi: 10.1155/2022/5660462 (PMC9363225; doi:10.1155/2022/5660462)

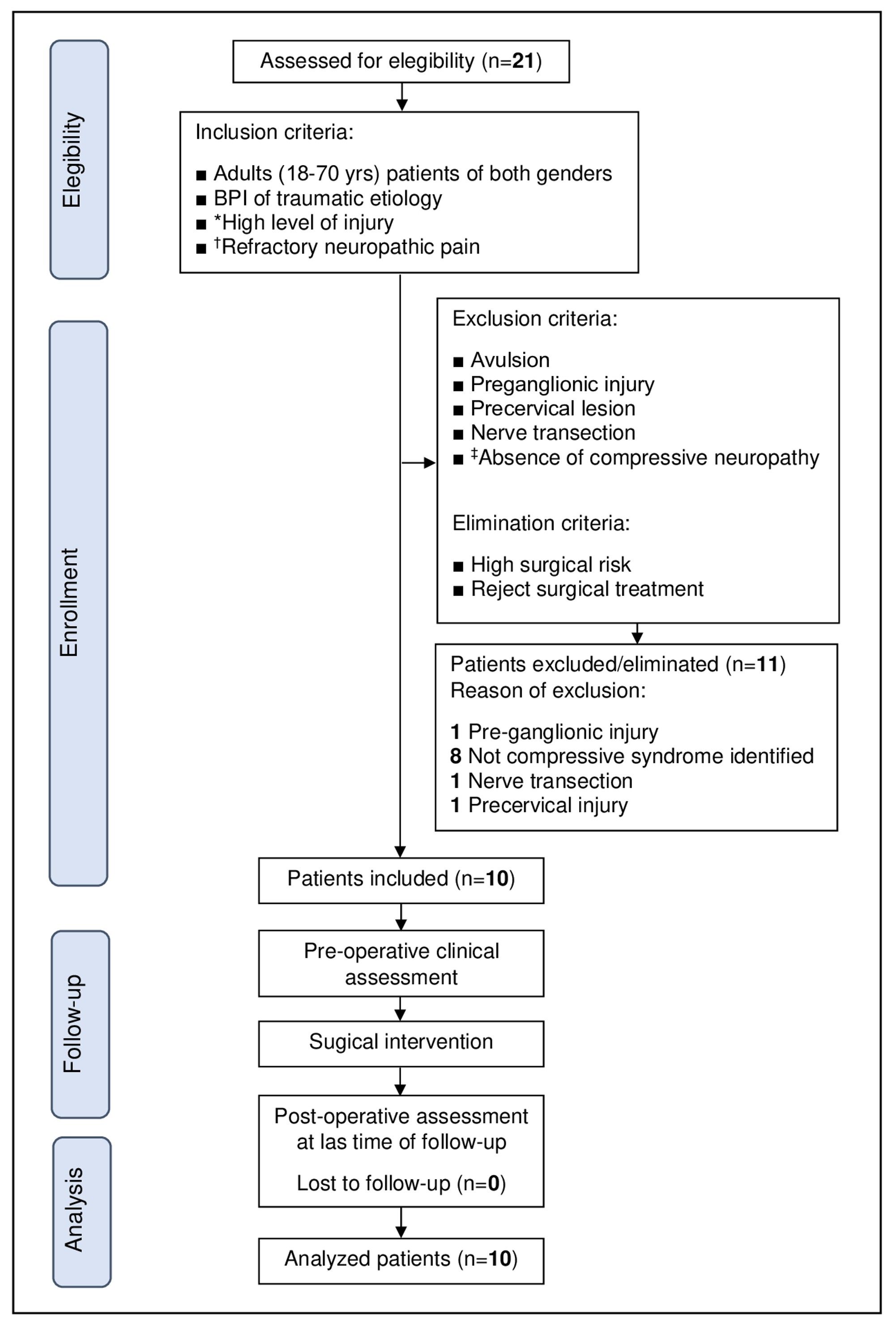

Supplement: Supplementary Materials — Supplementary 1. Supplemental Figure 1. CONSORT trial flow diagram. ∗Injury located in the proximal third of the upper extremity (before reaching the proximal third of the humerus). †Pain refractory to medical treatment with at least 2 different analgesic drugs during three months of management. ‡Compressive origin was determined through a preoperative electromyography study determined by a neurogenic pattern with positive fibrillations, polyphasic units, and an increase of firing rate. Supplementary 2. Supplemental Figure 2. A sample size calculation was carried out using the program: G∗ Power 3.1.9.7 for Windows XP. The sample size calculation was performed according to effect sizes for a Wilcoxon signed-rank test, using the results reported by Morgan R. et al. (2020) [8], study where they evaluated the effects of surgical neurolysis and open fasciotomy for pain relief in 21 patients with distal BPI, reporting changes in pain intensity according to VAS of 6.4 ± 2.5 2 ± 2.5 postoperatively, resulting in an effect size of 1,606. The sample size analysis resulted in a total number of required patients of 10 for a statistical power of 99%. s [file 5660462.f1.zip › Sup.Fig.1 (1).jpg]

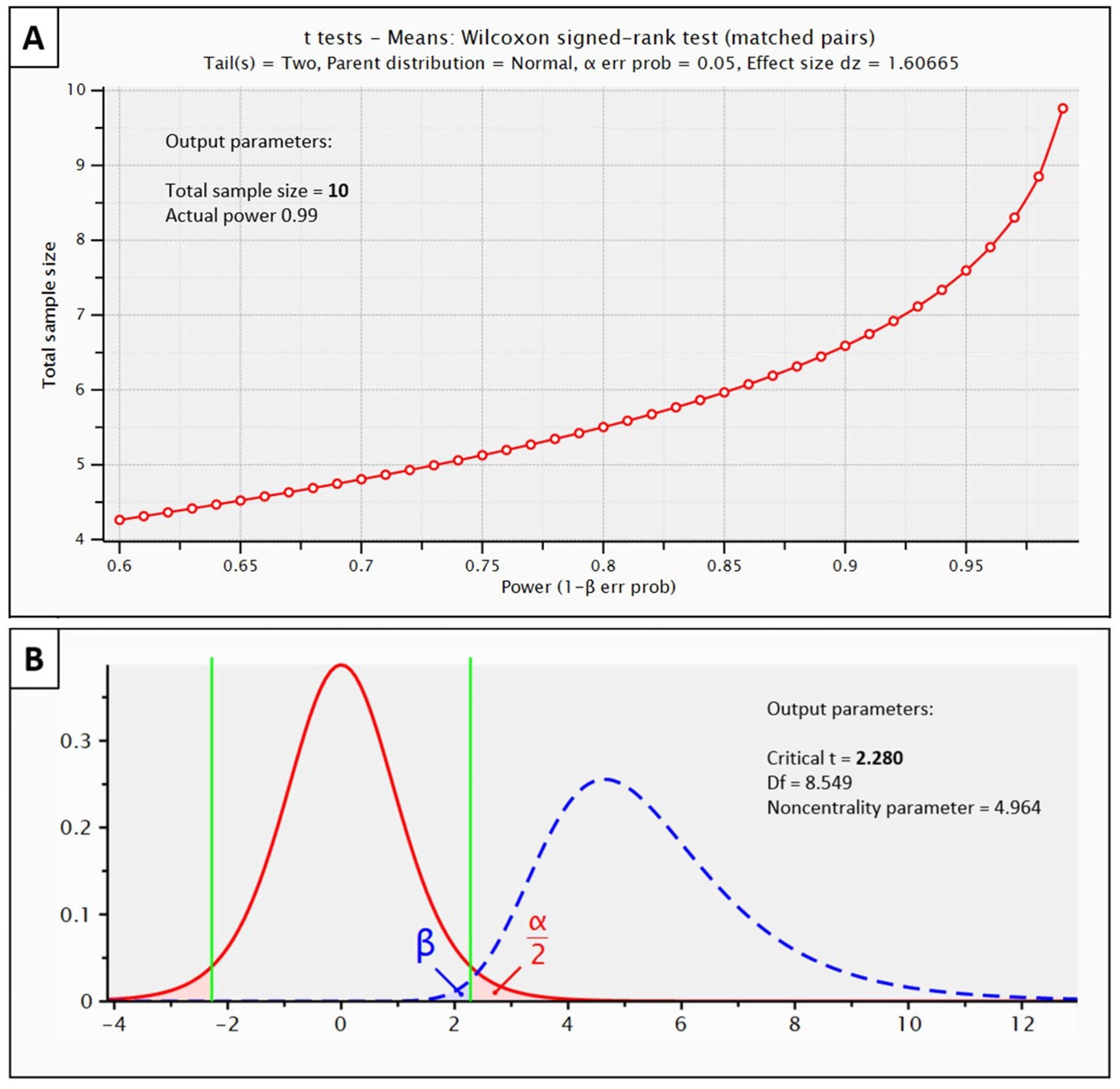

Supplement: Supplementary Materials — Supplementary 1. Supplemental Figure 1. CONSORT trial flow diagram. ∗Injury located in the proximal third of the upper extremity (before reaching the proximal third of the humerus). †Pain refractory to medical treatment with at least 2 different analgesic drugs during three months of management. ‡Compressive origin was determined through a preoperative electromyography study determined by a neurogenic pattern with positive fibrillations, polyphasic units, and an increase of firing rate. Supplementary 2. Supplemental Figure 2. A sample size calculation was carried out using the program: G∗ Power 3.1.9.7 for Windows XP. The sample size calculation was performed according to effect sizes for a Wilcoxon signed-rank test, using the results reported by Morgan R. et al. (2020) [8], study where they evaluated the effects of surgical neurolysis and open fasciotomy for pain relief in 21 patients with distal BPI, reporting changes in pain intensity according to VAS of 6.4 ± 2.5 2 ± 2.5 postoperatively, resulting in an effect size of 1,606. The sample size analysis resulted in a total number of required patients of 10 for a statistical power of 99%. s [file 5660462.f1.zip › Sup.Fig.2 (1).jpg]
